# Impact of a 3-Months Vegetarian Diet on the Gut Microbiota and Immune Repertoire

**DOI:** 10.3389/fimmu.2018.00908

**Published:** 2018-04-27

**Authors:** Chenchen Zhang, Andrea Björkman, Kaiye Cai, Guilin Liu, Chunlin Wang, Yin Li, Huihua Xia, Lijun Sun, Karsten Kristiansen, Jun Wang, Jian Han, Lennart Hammarström, Qiang Pan-Hammarström

**Affiliations:** ^1^BGI-Shenzhen, Shenzhen, China; ^2^Department of Laboratory Medicine, Karolinska Institutet, Stockholm, Sweden; ^3^HudsonAlpha Institute for Biotechnology, Huntsville, AL, United States; ^4^Laboratory of Genomics and Molecular Biomedicine, Department of Biology, University of Copenhagen, Copenhagen, Denmark; ^5^iCarbonX, Shenzhen, China

**Keywords:** vegetarian, gut microbiota, immunoglobulin, immune repertoire, B-cell, T-cell

## Abstract

The dietary pattern can influence the immune system directly, but may also modulate it indirectly by regulating the gut microbiota. Here, we investigated the effect of a 3-months lacto-ovo-vegetarian diet on the diversity of gut microbiota and the immune system in healthy omnivorous volunteers, using high-throughput sequencing technologies. The short-term vegetarian diet did not have any major effect on the diversity of the immune system and the overall composition of the metagenome. The prevalence of bacterial genera/species with known beneficial effects on the intestine, including butyrate-producers and probiotic species and the balance of autoimmune-related variable genes/families were, however, altered in the short-term vegetarians. A number of bacterial species that are associated with the expression level of IgA, a key immunoglobulin class that protects the gastrointestinal mucosal system, were also identified. Furthermore, a lower diversity of T-cell repertoire and expression level of IgE, as well as a reduced abundance of inflammation-related genes in the gut microbiota were potentially associated with a control group with long-term vegetarians. Thus, the composition and duration of the diet may have an impact on the balance of pro-/anti-inflammatory factors in the gut microbiota and immune system.

## Introduction

Diet is an important modulator of the immune system. While undernutrition can cause immunodeficiency and an increased mortality due to severe infections (1), overnutrition might lead to chronic inflammation and increased risk of immune disorders (2, 3). Calorie-restriction without malnutrition, on the other hand, has shown anti-inflammatory effects in several murine studies (4). An immune response requires nutrients for rapid protein synthesis and cell proliferation. Furthermore, certain vitamins and trace elements are needed for specific immunological functions (5). Many lipids and fatty acids also have immune-regulatory effects (2). Moreover, the consumption of certain types of foods, such as processed meat and, to a lesser extent, unprocessed red meat, could have adverse effects on the immune system, including a predisposition to immune/inflammation-related disorders, such as type-2 diabetes (T2D) (6). On the other hand, a vegetarian diet, which is often rich in vitamins and fibers, seems to reduce the risk of development of obesity (7), asthma (8), and atopic dermatitis (9).

The human gut is populated with a diverse range of symbiotic bacterial species, collectively termed the gut microbiota. A healthy intestine is dependent on a balance between pro- and anti-inflammatory signals to be able to provide tolerance to beneficial bacteria and meanwhile fight against intestinal pathogens. Consequently, the gut microbiota and immune system have co-evolved and are dependent on each other for their functions (10). A deregulated gut microbiota seems to be associated with many immune-related disorders, including inflammatory bowel disease (IBD), diabetes, and allergy (11–13), as well as cancers (14). The importance of the gut microbiota for a normal immune system has furthermore been demonstrated by several studies in germfree animals, which show impaired immune functions, including poorly developed intestinal lymphoid structures, low levels of CD4^+^ T-cells, and deficiency of secretory immunoglobulin (Ig) A in the gut (10). IgA is the main Ig class expressed in the intestine and its presence, as well as its diversification and quality, might in turn regulate the complexity of the microbiota (15–17).

A more indirect effect of the diet on the immune system is thus through its modulation of the gut microbiota. Several studies have shown how dietary factors, including long-term diets with a high content of either protein and animal fat or carbohydrates (18), short-term vegetarian or meat diets (19), change from low fat/high plant polysaccharide to high fat/high sugar diet (20), protein (21), and fiber (22) content, or energy restricted diet (23) can influence the gut microbiota to various degrees.

High-throughput next-generation sequencing (NGS) technologies have allowed the establishment of a catalog of human gut microbial genes (24–27). This effort has enabled studies of the composition of the gut microbiota and its association with human diseases, including immune disorders (28, 29). Furthermore, these technologies have also been applied for studies on the B-cell receptor (BCR) and T-cell receptor (TCR) compositions (30, 31). The sum of functional BCRs and TCRs at a given time, referred to as the immune repertoire, is shaped by both genetic and environmental factors (30, 31). In this report, we have studied the impact of a 3-month lacto-ovo-vegetarian diet on the gut microbiota and immune repertoire using NGS technologies.

## Materials and Methods

### Study Design and Sample Preparation

**Figure 1:**
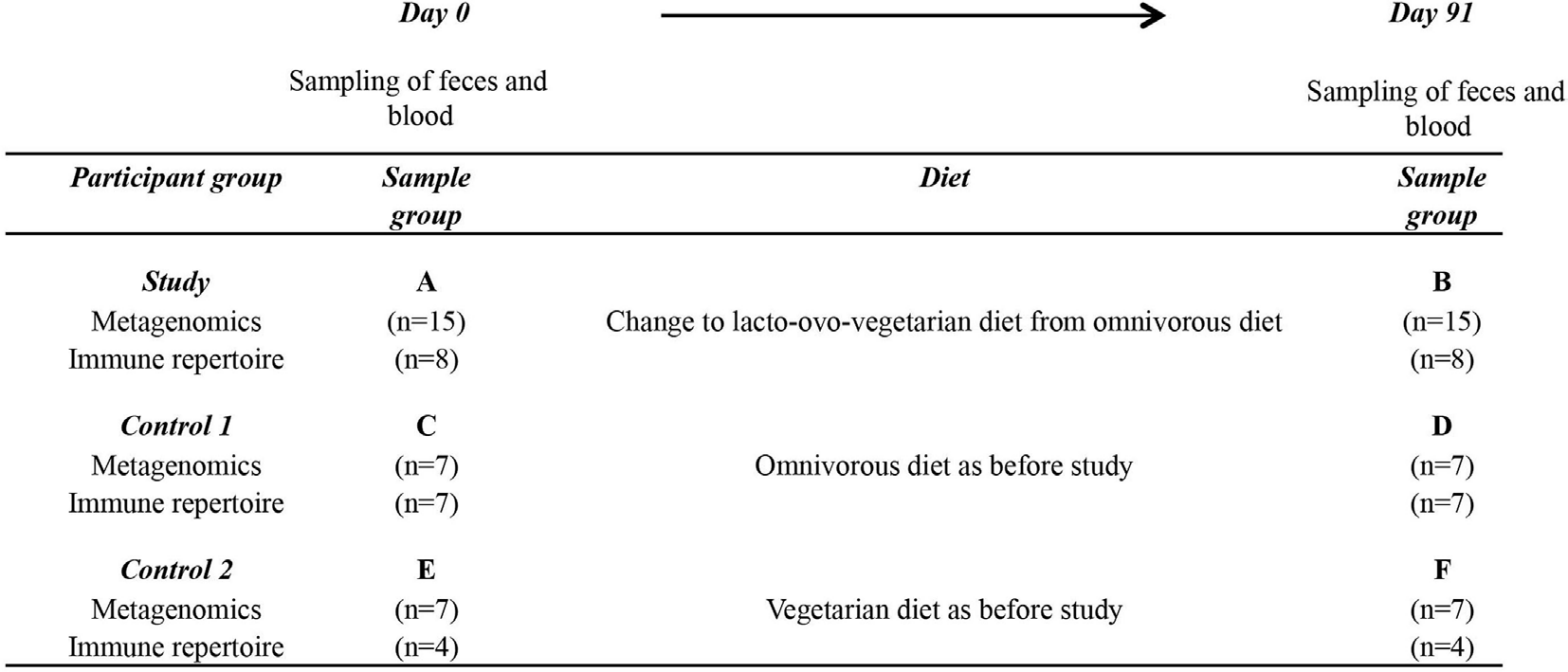
Study design. The type of samples, at which time points the samples were collected and the number of individuals included in each group are specified. The change in diet category for each group during study period is also indicated in the figure.

Fifteen healthy volunteers (study group), who normally consume an omnivorous diet, were recruited to the study (Figure 1). They changed to a lacto-ovo-vegetarian diet for 3 months. In addition, seven healthy omnivorous volunteers (control group 1) and seven healthy long-term vegetarians (lacto-ovo-vegetarian diet, control group 2) were recruited. The participants had not taken antibiotics 3 months before the study nor during the study period. Blood and fecal samples were collected and weight was measured days 0 and 91 for all individuals.

RNA was purified from PBMCs using the RNeasy Kit (Qiagen, Hilden, Germany). Fecal samples were stored at −80°C immediately after sampling and fecal microbial DNA was isolated by the PowerLyzer PowerSoil DNA Isolation Kit (MO BIO, Carlsbad, CA, USA). Detailed sample information of all sample donors was collected and presented in Table S1 in Supplementary Material. All study subjects have signed a consent to participate in the study. The study was carried out in accordance to the Karolinska Institutet regulations and approved by the institutional review board at Karolinska Institutet.

### Immune Repertoire Sequencing

The immune repertoires for the BCRs and TCRs were semi-quantitatively amplified by an amplicon-rescued multiplex nested PCR method using primers specific for the various *V*- and *C*-genes (iRepertoire, Huntsville, AL, USA) (Figure S1 in Supplementary Material). In brief, 1 µg of RNA was added to a reaction containing either HTIvc (TCR) or HTBIvc (BCR) primers with different barcodes (iRepertoire) and reagents from the OneStep RT-PCR kit (Qiagen). Subsequently, 2 µl of PCR1 products were added to a reaction with Illumina common primers (iRepertoire) and reagents from Multiplex Master Mix kit (Qiagen). The PCR2 products were gel purified using QIAquick gel extraction Kit (Qiagen). Equal amounts of DNA with different barcodes were pooled and sequenced at the sequencing center of the HudsonAlpha Institute (Illumina Hiseq 2000).

The method for alignment of the sequences has been described previously (32). Briefly, sequences were aligned to their germline V-, D-, and J-genes by the Smith–Waterman algorithm and assigned according to the IMGT/GENE-DB reference directory. The D50-value was calculated by dividing *X* with *Y* and multiplying with 100, where *X* = number of unique complementary determining regions 3 (CDR3s) that account for the cumulative 50 percent of the total sequences, *Y* = number of unique CDR3s. To obtain the delta index, the sequences from one sample were first normalized to 10 × 10^6^ reads. These fractions were then compared between the samples from days 0 and 91, from the same individual, and the difference was calculated and the CDR3s were subsequently sorted and ranked according to this value. Next, the sum of all frequency differences of the 1,000 most common CDR3 clones was calculated and a number between 0 (identical samples) and 2 (samples that share no CDR3s) was obtained.

### DNA Library Construction and Metagenome Sequencing

DNA library construction was performed following the manufacturer’s instruction (Illumina). A similar workflow as described previously (25) was used to perform cluster generation, template hybridization, isothermal amplification, linearization, blocking and denaturation, and hybridization of the sequencing primers. Paired-end (PE) library with insert size of 350 bp for each sample was constructed, followed by high-throughput sequencing to obtain around 60 million PE reads. The read length for each end is 100 bp. The metagenomic sequencing data is available at the European Bioinformatics Institute (EMBL-EBI). BioProject Accession: PRJEB26089, SRA Accession: ERP108062.

### Data Profile Construction

High quality reads were mapped to the recently published reference gut integrated gene catalog (IGC) (26) (identity > = 95%), and we obtained a gene catalog that contained 5,213,654 genes. Computation of the relative gene abundance was performed as previously described (25). The gene catalog was taxonomically annotated using reference genomes of 3,449 bacteria and archaea, and functionally annotated according to Kyoto Encyclopedia of Genes and Genomes (KEGG). Relative abundance of phylum/genus/species/KEGG orthologous (KO) was estimated by summing the abundance of genomes belonging to that phylum/genus/species/KO, respectively.

### Identification of Enterotypes and Estimation of Within-Sample (Alpha) Diversity and Between Sample (Beta) Diversity

Enterotypes based on genus profile were identified using similar method as described previously (33). In this study, samples were clustered using the Jensen–Shannon distance and illustrated by a principal component analysis (PCA) graph that was implemented in “ade4” package in R software (34).

Based on the genus/species/gene/KO profiles, the within-sample (alpha) diversity was calculated to estimate the genus/species/gene/KO richness of a sample using Shannon index. The between sample (beta) diversity was furthermore calculated, using Jensen–Shannon distance, to estimate the difference of the genus/species/gene/KO richness between different samples.

### Permutational multivariate analysis of variance (PERMANOVA)

Permutational multivariate analysis of variance was used to assess the effect of different covariates, such as enterotypes, gender, body mass index, age, immune indexes, and diet history on the metagenome gene profile. The analysis was performed using the method implemented in the R package—“vegan” (35), and the permuted *p*-*value* was obtained by 10,000 permutations.

### Reporter Score for Pathways/Modules

Gene functional analysis was performed based on KO profile. Significant modules and pathways (the KEGG Class Level 3) were identified using a reporter feature algorithm (36) based on the pathway-KO and module-KO analyses. One-tail Wilcox rank-sum test was first performed on all the KOs and adjusted for multiple-test using FDR controlling. Then, the reporter score for each pathway/module was computed using $p.a_{{\text{KO}}_{i}}$ based on the module-KO or pathway-KO relations as:

$$Z_{{\text{KO}}_{i}}=\theta^{-1}(1-p\cdot a_{{\text{KO}}_{i}})$$

$$Z_{\text{pathway}}=\frac{1}{\sqrt{k}}\sum Z_{{\text{KO}}_{i}}$$

*k* denotes the number of KO involved in the pathway. Third, the background distribution of *Z*_pathway_ was corrected by subtracting the mean –*k* and dividing by the standard deviation (–*k*) of the aggregated *Z* scores of 1,000 sets of *k* KO chosen randomly from the whole metabolic KO network by:

$$Z_{\text{ajusted pathway}}=\frac{Z_{\text{pathway}}-{\_}_{k}}{{\_}_{k}}$$

The *Z*_adjusted pathway_ was used as the final reporter score for evaluating the degree and direction of enrichment of specific pathway/module. A reporter score of more than 1.96 or less than −1.96 was used as the detection threshold for significantly altered pathway.

## Results

### Sequencing the Metagenomes

Twenty-nine healthy volunteers were enrolled as three groups (study, control 1, and control 2 groups) and samples were collected on days 0 (A, C, and E) and 91 (B, D, and F), respectively (Figure 1; Table S1 in Supplementary Material). The participants in the study group (*n* = 15) changed to a lacto-ovo-vegetarian diet for 3 months (from this point onward referred to as a short-term vegetarian diet), whereas the members of control 1 (*n* = 7) and control 2 (*n* = 7) groups continued on their usual omnivorous or vegetarian diet, respectively. To investigate the effect of various diets on gut microbiota, we performed metagenomics shotgun sequencing on samples collected at two time points from all participants. On average, 57,663,666 raw reads and 56,878,337 high quality reads per sample were generated and these were similar among the different groups (Table S2 in Supplementary Material). The high quality reads were subsequently mapped to the 9.9 million IGC and 5,213,654 genes were identified in our dataset. These were taxonomically annotated using reference genomes of 3,449 bacteria and archaea (37), and functionally annotated according to the KEGG.

### Impact of the Short-Term Vegetarian Diets on Metagenome

**Figure 2:**
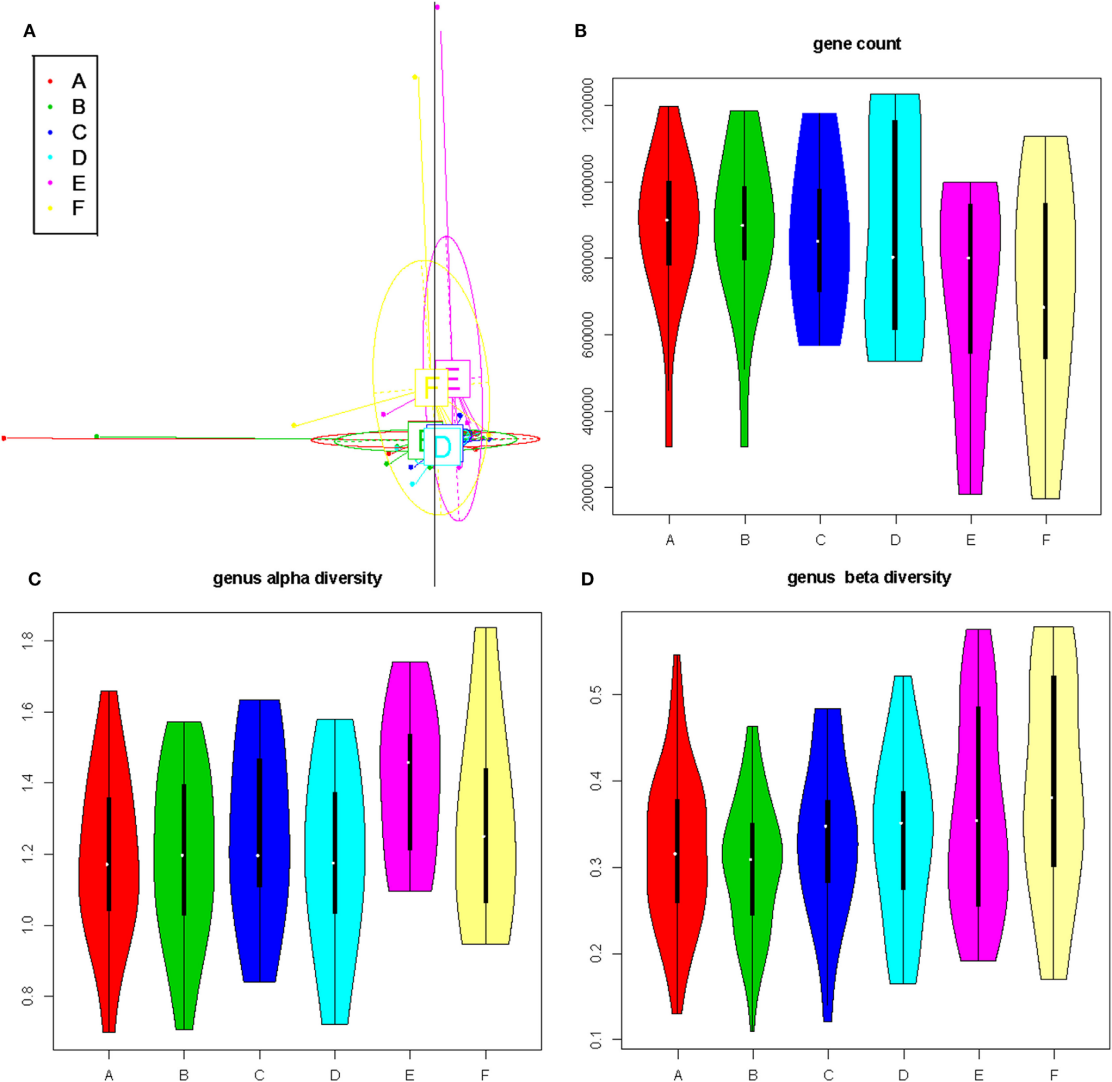
The impact of diet pattern on general composition, richness, and diversity of the gut microbiota. **(A)** Principal component analysis based on the gut bacterial gene profile. **(B)** Violin plots showing the richness at the gene level for each subgroup. **(C,D)** Violin plots showing the alpha- and beta-diversity at the genus level, for each subgroup.

To obtain an overview of the composition of the microbiota, we first conducted an enterotype analysis on the genus level (33). Three enterotypes were identified in our samples (Figure S2A in Supplementary Material). A PCA showed that these three enterotypes were primarily made up by several highly abundant genera, including *Alistipes, Bacteroides, Prevotella*, and *Akkermansia* (Figure S2B in Supplementary Material). No significant relationship between enterotype and diet pattern was, however, identified. PCA was furthermore performed based on gene profile and demonstrating that samples from different groups clustered together (Figure 2A).

We next investigated the richness and evenness of the gut microbiota in all samples at the genus, species, and gene levels. No significant difference was observed in gene count or alpha diversity (Shannon index) at all levels between the two time points in any groups (Figures 2B,C and data not shown). The beta diversity, however, accessed by using Jensen–Shannon divergence, showed a trend for reduction after changing to the short-vegetarian diet, and reached significance at the genus and species level (A vs. B subgroup, Figure 2D; Figure S3 and Table S3 in Supplementary Material). Thus, the general composition of gut microbiota did not seem to be associated with the diet pattern, but there might be specific alterations associated with the change to the short-term vegetarian diet, especially on the genus and species levels.

**Figure 3:**
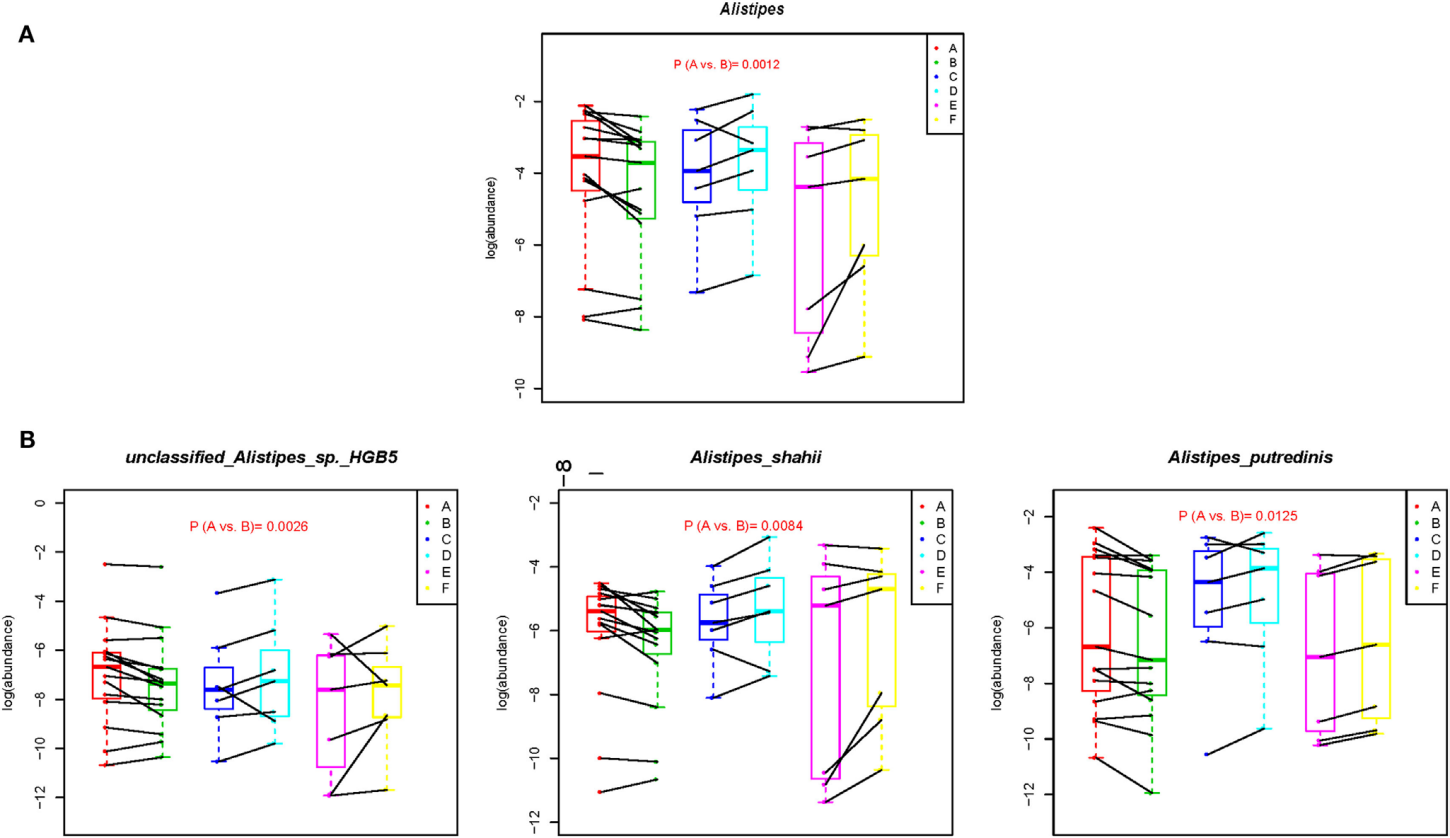
Metagenomic biomarkers associated with short-term vegetarian diet. The relative abundance (log) of selected genera **(A)** and species **(B)** that were significantly different between A and B subgroups were plotted for each subgroup. Wilcoxon signed-rank test was used for the statistical calculation. The complete dataset is presented in Tables S4 and S5 in Supplementary Material.

We next directly compared the bacterial profiles from before- (A subgroup) and after- (B subgroup) samples to search for biomarkers that might be associated with the alteration of dietary pattern. At the phylum level, no significant change was observed between the A and B subgroups. However, seven genera were significantly associated with change to the short-term vegetarian diet (Table S4 in Supplementary Material). Specifically, *Alistipes*, a high abundance and bile-tolerant microorganism, which is enriched after a short-term animal-based diet (19), was significantly reduced in the B subgroup samples (Figure 3A). Accordingly, the abundances of three species belonging to *Alistipes*, unclassified *Alistipes sp*. HGB5, *Alistipes shahii*, and *Alistipes putredinis* were decreased after the short-term vegetarian diet (Figure 3B; Table S5 in Supplementary Material). In addition, the abundance of nine species was significantly associated with the change in diet. For example, the abundance of *Roseburia inulinivorans, Ruminococcus lactaris, Lactobacillus plantarum* (*L. plantarum*) and *Streptococcus thermophilus*, or *Proteus mirabilis* was increased and reduced, respectively, in the B subgroup samples (Table S5 in Supplementary Material).

To search for biomarkers that might be associated with a long-term vegetarian diet, we compared the gut bacterial profiles among controls and observed a list of genera (*n* = 32) or species (*n* = 55) that differed significantly between the two control groups (*p*-value <0.05 using Wilcoxon rank-sum test) (Table S6 in Supplementary Material). *Bacteroides finegoldii* is found to be enriched in the control group 1, the omnivores. This species belongs to a group of bacteria that grow in the presence of bile (38) and has been reported to be associated with colon cancer (39). Species enriched in the long-term vegetarians included the butyrate-producing *Peptoniphilus duerdenii* and *Clostridium symbiosum* (40), the H2-utilizing acetogen *Blautia hydrogenotrophica* (formally known as *Ruminococcus hydrogenotrophicus*), which contributes to the degradation and fermentation of dietary fibers (41).

### Short- and Long-Term Vegetarian Diet-Associated Functional Changes in the Microbiome

**Figure 4:**
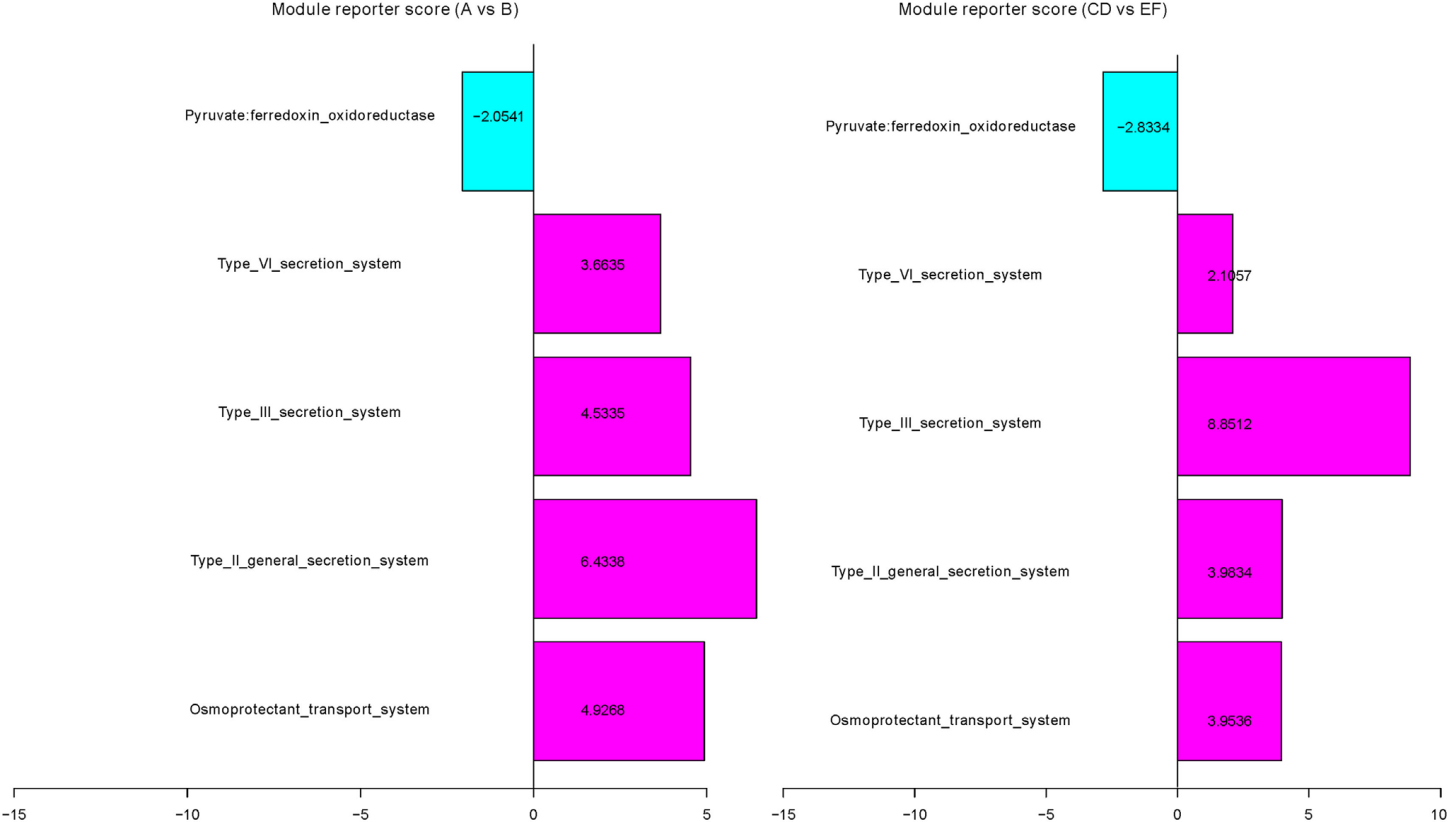
Selected Kyoto Encyclopedia of Genes and Genomes orthologous (KO) modules associated with short- and long-term vegetarian diets. The relative abundances of KO modules were compared between A and B subgroups, between control 1 and control 2 groups (for complete dataset, see Table S7 and Figure S4 in Supplementary Material). Blue, enriched in subgroup A or control group 1; pink, enriched in subgroup B or control group 2.

We next studied the impact of the diet on the microbiota based on KO groups. A reporter score (36) was subsequently calculated to estimate the significance of variation at the functional module level among different groups (Table S7 and Figure S4 in Supplementary Material). Several modules show a similar trend of enrichment or depletion in the short- and long-term vegetarians. For example, the module of the osmoprotectant transport system (M00209), mediating the cellular uptake of choline, carnitine, and betaine, which are mainly from red meat (25, 37), was significantly enriched in omnivorous individuals (Figure 4). Similarly, modules of type II, II, and IV general secretion systems (M00331, M00332, and M00334), associated with cholera toxin and intracellular toxins (42), were also significantly enriched in omnivorous individuals (Figure 4). Pyruvate:ferredoxin oxidoreductase (M00310), which is involved in short-chain fatty acids production, was enriched in both short- and long-term vegetarians.

**Figure 5:**
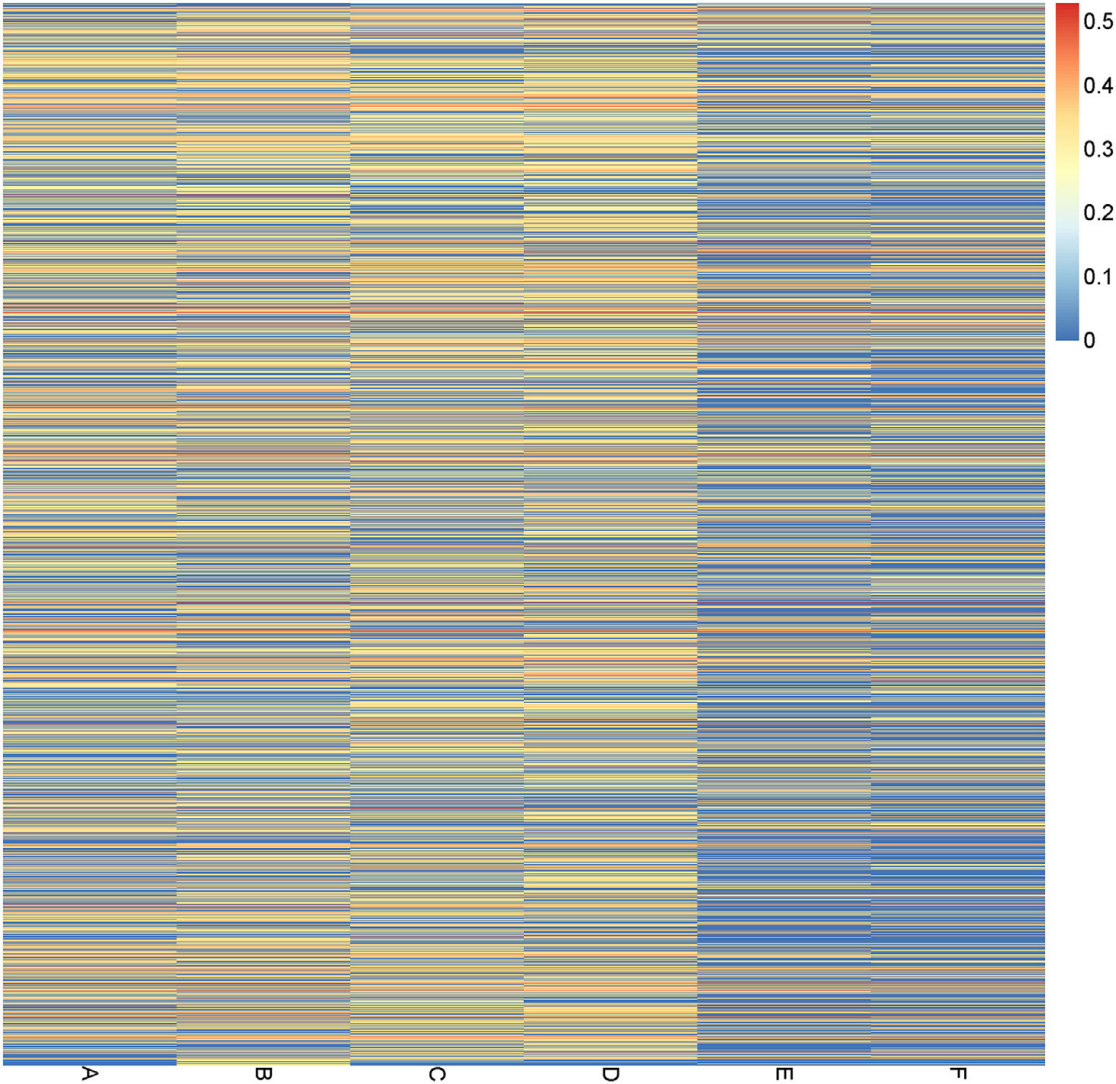
Abundance of bacterial genes in the lipopolysaccharide-related pathways in different subgroups. Each line for one gene, and the value (from 0 to 1) related to its average abundance in each subgroup is shown as a heatmap.

We next compared the abundance of genes belonging to pathways and modules related to lipopolysaccharide (LPS) biosynthesis (map00540 and M00063), which are known to be involved in inflammation, among the study group and two control groups. The heatmap of these pathways/modules (Figure 5; Figure S5 in Supplementary Material) showed a clear pattern of low abundance of LPS related genes in the samples from control group 2, but no significant difference between the A and B subgroups was observed. Thus, the long-term, but not the short-term, vegetarian diet may have an anti-inflammatory effect based on the functional pathway analysis of the gut microbiome.

### High-Throughput Sequencing of the BCR and TCR Repertoires

We next studied the B- and T- repertoires in a subset of patients (38 samples from 19 patients, Figure 1; Table S1 in Supplementary Material). The CDR3 regions, which span the V(D)J recombination junction and represent the most diverse parts of BCR [Ig heavy (H) chain] or TCR (beta chain), were amplified from peripheral blood samples by a nested PCR strategy using primers specific for the different *variable* (*V)*- and *constant (C)*-genes (Figure S1 in Supplementary Material). The PCR products were sequenced on the Illumina HiSeq 2000 platform. On average, 1,853,396 and 5,300,946 reads were obtained from each sample by sequencing the *IGH* and *TCR beta* chain, respectively (Table S8 in Supplementary Material).

### Impact of Diet Pattern on the Diversity of Immune System

**Figure 6:**
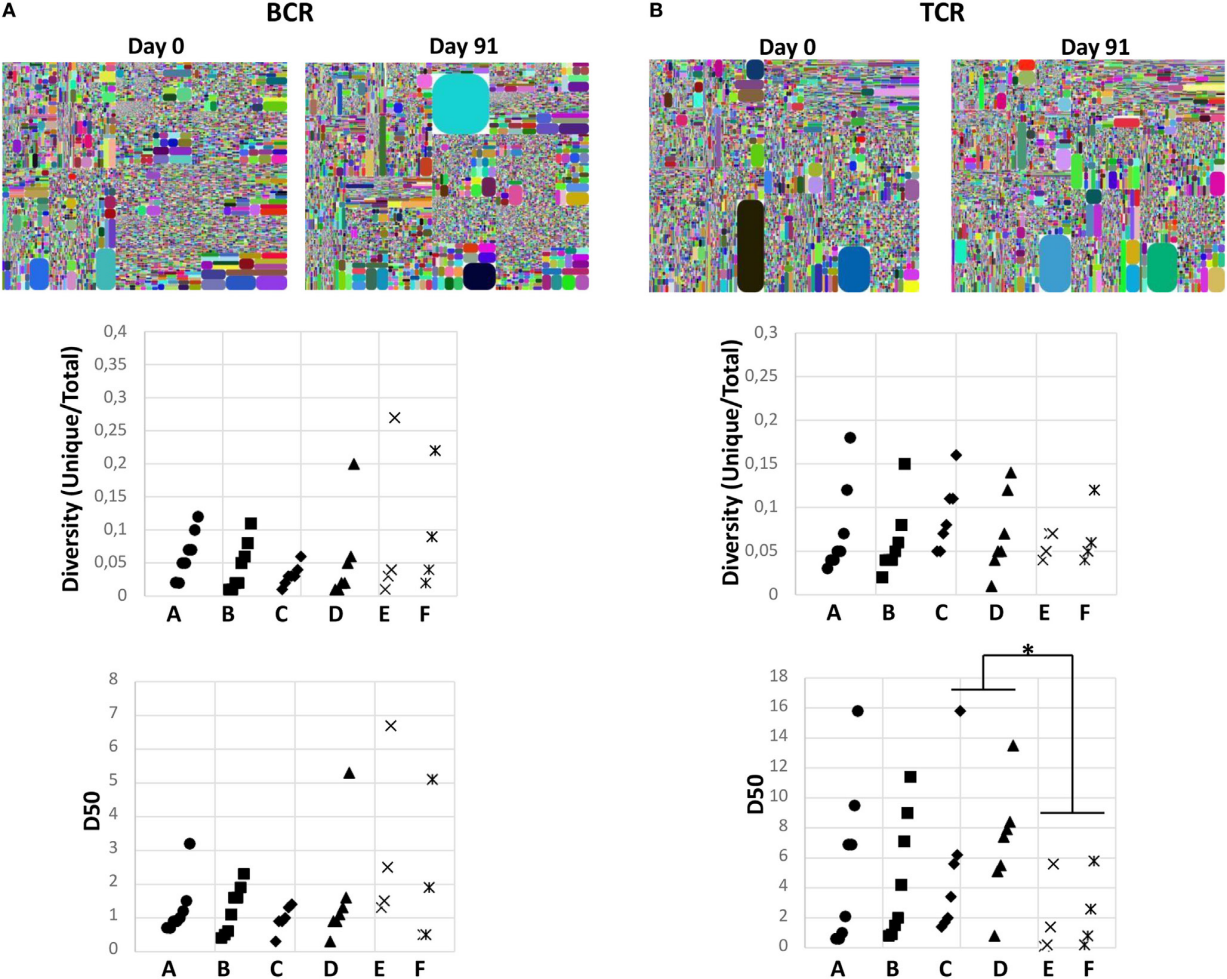
Impact of diet pattern on the diversity of immune system. Diversities of B-cell receptor **(A)** and T-cell receptor **(B)** were analyzed in all study subjects from the different groups. Representative tree map from samples collected at days 0 and 91 from one individual in the study group are shown on top. Each dot represents one unique complementary determining regions 3 (CDR3)-V-J combination, whereas the size illustrates the number of sequences of each CDR3-V-J combination. Changes in D50 values and diversity (unique/total CDR3s) between days 0 and 91 in each group are shown in the middle and at the bottom panels, respectively. Each line represents one individual. Statistical calculations were performed by Wilcoxon signed-rank test. *p < 0.05.

To investigate whether the change in diet had any global effect on the immune system, the diversities of BCR and TCR repertoires were measured in all samples. Diversity, defined as unique CDR3s divided by the number of total sequences, was first analyzed and no significant changes were observed between the two time points in the study or control groups (Figure 6). Furthermore, the D50 values, which defines the percent of unique B- or T-cell clones that account for the cumulative 50% of the total CDR3s in a given sample, was calculated. This is a measurement of the evenness of the B- or T-cell clones, where a low D50-value indicates a repertoire skewed by large clonal expansions. Again there was neither significant change between days 0 and 91 in the study nor in the control groups (Figure 6; Table 1; Table S9 in Supplementary Material). However, the D50-value for TCR repertoire was significantly lower in the long-term vegetarians (control group 2) compared to the omnivores (control group 1), whereas an opposite trend was observed for BCR repertoire (Table 1). Furthermore, no significant changes in delta index, which reflects the percentage of repertoire turnover, were observed in any group (Table 1). Thus, the change to a vegetarian diet for a 3-month period did not seem to have a major effect on the overall diversity of the immune repertoire, but a long-term vegetarian diet might be associated with a lower diversity of TCR repertoire.

**Table 1:** Mean values for age, body mass index, and selected immune parameters in different subgroups included in immune analysis^a^.

Parameter^b^	A	B	C	D	E	F
Age (years)	39.0 ± 10.1		34.7 ± 10.4		33.5 ± 7.4	
BMI	23.9 ± 2.8	23.4 ± 2.4	21.7 ± 3.0	21.8 ± 3.0	21.1 ± 3.5	21.0 ± 3.3
**Immune parameters**						
D50-value (BCR)	1.3 ± 0.8	1.3 ± 0.7	1.1 ± 0.4	1.6 ± 1.7	3.0 ± 2.5	2.0 ± 2.2
D50-value [T-cell receptor (TCR)]	5.4 ± 5.4	4.6 ± 4.1	5.2 ± 5.1	6.9 ± 3.9	1.8 ± 2.6	2.4 ± 2.5
Delta index [B-cell receptor (BCR)]	0.54 ± 0.23		0.69 ± 0.18		0.49 ± 0.06	
Delta index (TCR)	0.12 ± 0.06		0.14 ± 0.07		0.13 ± 0.05	
**IgD (%)c**	16.8 ± 2.3	14.9 ± 2.3	15.1 ± 4.7	14.8 ± 2.2	15.7 ± 1.3	13.5 ± 3.0
IgM (%)	59.7 ± 7.7	56.8 ± 11.6	51.1 ± 10.5	54.6 ± 9.1	59.2 ± 4.1	63.6 ± 8.4
IgG (%)	6.0 ± 1.6	5.5 ± 1.3	7.0 ± 0.5	6.6 ± 0.8	5.5 ± 0.8	5.1 ± 1.2
IgA (%)	10.7 ± 6.6	15.6 ± 13.5	19.9 ± 13.2	17.1 ± 3.3	13.2 ± 3.3	11.4 ± 4.1
IgE (%)	2.1 ± 1.0	1.8 ± 0.7	2.5 ± 0.5	2.6 ± 0.5	1.7 ± 0.5	1.9 ± 0.7
Unknown (%)	4.7 ± 0.9	5.3 ± 1.2	4.4 ± 0.8	4.4 ± 2.4	4.9 ± 2.4	4.6 ± 1.8
*IGHV1* (%)^d^	9.5 ± 3.3	9.4 ± 2.5	7.3 ± 1.7	7.0 ± 0.9	7.2 ± 2.2	8.2 ± 2.3
*IGHV2* (%)	1.0 ± 0.1	0.9 ± 0.3	1.0 ± 0.2	1.0 ± 0.2	0.8 ± 0.2	1.0 ± 0.1
*IGHV3* (%)	45.7 ± 3.8	39.7 ± 5.9	49.7 ± 5.3	49.5 ± 4.1	52.2 ± 9.1	53.2 ± 3.8
*IGHV4* (%)	34.4 ± 5.2	35.3 ± 4.5	32.5 ± 3.6	32.3 ± 2.9	33.2 ± 7.9	30.9 ± 5.3
**IGHV5 (%)**	7.4 ± 2.1	12.0 ± 5.6	7.2 ± 2.4	7.9 ± 2.6	4.4 ± 1.4	4.3 ± 1.1
*IGHV6* (%)	1.9 ± 0.5	2.5 ± 0.9	2.2 ± 1.1	2.1 ± 0.8	1.8 ± 0.3	1.6 ± 0.3
*IGHV7* (%)	0.2 ± 0.3	0.3 ± 0.3	0.2 ± 0.2	0.2 ± 0.2	0.4 ± 0.4	0.3 ± 0.4
*IGHV3-d* (%)	0.1 ± 0.0	0.1 ± 0.1	0.2 ± 0.1	0.1 ± 0.0	0.1 ± 0.0	0.1 ± 0.0
*IGHV3-9* (%)	0.6 ± 0.8	0.3 ± 0.1	0.3 ± 0.1	0.2 ± 0.1	0.5 ± 0.2	0.4 ± 0.1
**IGHV3-15 (%)**	1.6 ± 0.3	1.3 ± 0.3	1.8 ± 0.3	1.8 ± 0.5	1.9 ± 0.5	1.8 ± 0.3
*IGHV3-23* (%)	6.1 ± 1.5	5.7 ± 1.6	6.9 ± 1.0	7.4 ± 1.2	8.3 ± 2.2	8.1 ± 1.8
**IGHV3-33 (%)**	3.3 ± 0.5	3.0 ± 0.5	3.5 ± 0.4	3.3 ± 0.6	3.9 ± 1.1	4.2 ± 0.9
**IGHV3-64 (%)**	6.5 ± 1.8	5.7 ± 1.6	7.4 ± 1.5	7.2 ± 1.5	7.4 ± 1.4	7.6 ± 0.9
**IGHV3-74 (%)**	2.2 ± 0.3	1.8 ± 0.3	2.3 ± 0.4	2.1 ± 0.4	2.3 ± 0.8	2.4 ± 0.6
*IGHV4-34* (%)	10.5 ± 3.5	10.7 ± 3.2	10.2 ± 1.6	10.0 ± 1.6	10.6 ± 2.5	10.0 ± 2.4
*IGHV4-39* (%)	7.2 ± 1.8	7.1 ± 1.8	5.9 ± 1.5	5.7 ± 1.6	5.0 ± 0.5	5.0 ± 0.5
**IGHV5-51 (%)**	3.9 ± 1.1	5.9 ± 3.0	3.8 ± 1.3	4.2 ± 1.4	2.4 ± 0.8	2.7 ± 0.6
**IGHV5-a (%)**	3.4 ± 1.0	5.5 ± 3.1	3.4 ± 1.2	3.7 ± 1.2	2.0 ± 0.7	2.3 ± 0.5
*TRBV3-1* (%)	1.2 ± 1.3	1.2 ± 1.5	3.2 ± 0.5	3.0 ± 0.5	0.8 ± 1.5	0.8 ± 1.5
*TRBV5-8* (%)	0.2 ± 0.1	0.2 ± 0.1	0.2 ± 0.1	0.2 ± 0.1	0.2 ± 0.0	0.2 ± 0.0
**TRBV7-4 (%)**	0.029 ± 0.008	0.033 ± 0.007	0.029 ± 0.008	0.025 ± 0.006	0.047 ± 0.016	0.045 ± 0.019
*TRBV9* (%)	1.0 ± 0.2	1.1 ± 0.3	1.1 ± 0.3	1.0 ± 0.3	0.8 ± 0.2	0.8 ± 0.2
*TRBV16* (%)	0.1 ± 0.0	0.1 ± 0.0	0.1 ± 0.0	0.1 ± 0.0	0.1 ± 0.0	0.1 ± 0.0

*^a^Thirty-eight samples from days 0 and 91, respectively, from the study (*n* = 8, *n* = 8), control 1 (*n* = 7, *n* = 7), and control 2 (*n* = 4, *n* = 4) groups were included for immune analysis*.

*^b^Parameters that are significantly different between subgroups A and B are indicated in bold letters (Wilcoxon signed-rank test). The statistics for the complete data set are presented in Table S9 in Supplementary Material*.

*^c^Ig frequencies are calculated based on unique Ig–CDR3–C–J combinations*.

*^d^V gene usage is calculated based on unique CDR3–V–J combinations*.

*BMI, body mass index*.

The average length of CDR3 regions, as well as the frequency of shared CDR3s was largely similar between days 0 and 91 in all groups. The usage of specific *V* genes, such as *IGHV3-3, IGHV3-64* and *IGHV3-74* (43), *IGHV4-34* (44), *IGHV5-51* (45), and *TCR beta* (*TRB*) *TRBV14* and *TRBV16* (46) has been correlated with CDR3s binding to certain pathogenic antigens and to autoimmune and inflammatory disorders (47). The *IGH* and *TRB V* gene frequencies were largely similar between the two time points. However, the frequency of the *IGHV5* family (containing *IGHV5-a* and *IGHV5-51*), as well as *IGHV3-64, IGHV3-74, IGHV3-15, IGHV3-33*, and *TRBV7-4* were significantly altered in the study group (Table 1). In addition, the *IGHV5* and *IGHV7* families, as well as *IGHV3-9, IGHV3-d, IGHV4-39* and *TRBV3-1, TRBV9, TRBV16*, and *TRBV7-4* showed significantly different frequencies between the two control groups (Table 1).

The Ig isotype frequencies, identified by BCR sequencing, were subsequently examined. Notably, the study group showed a higher expression of IgA at day 91 compared to day 0 (15.6 vs. 10.7%), albeit not to a significant degree, whereas the frequency of other Ig isotypes was reduced (Table 1). Furthermore, the long-term vegetarians had significantly higher levels of IgM, but lower levels of IgG and IgE as compared to the omnivorous control group (Table 1; Table S9 in Supplementary Material).

### Interplays Between the Immune System and the Gut Microbiota

Permanova analyses were subsequently used to assess whether certain phenotypes, including the diet pattern and immune indexes, have significant impact on the gut microbiota. As shown in Table S10 in Supplementary Material, gender, age, BMI, enterotype, and long-term diet pattern (meat-based or plant-based diets) significantly influenced the microbial gene profile in the gut. Furthermore, many immune parameters, including the expression of certain Ig isotypes (IgD, IgM, IgG, and IgA), several *IGH* and *TRB V* families/genes and the diversity of TCR, also had a significant association with the gut microbiota (Table S10 in Supplementary Material).

**Figure 7:**
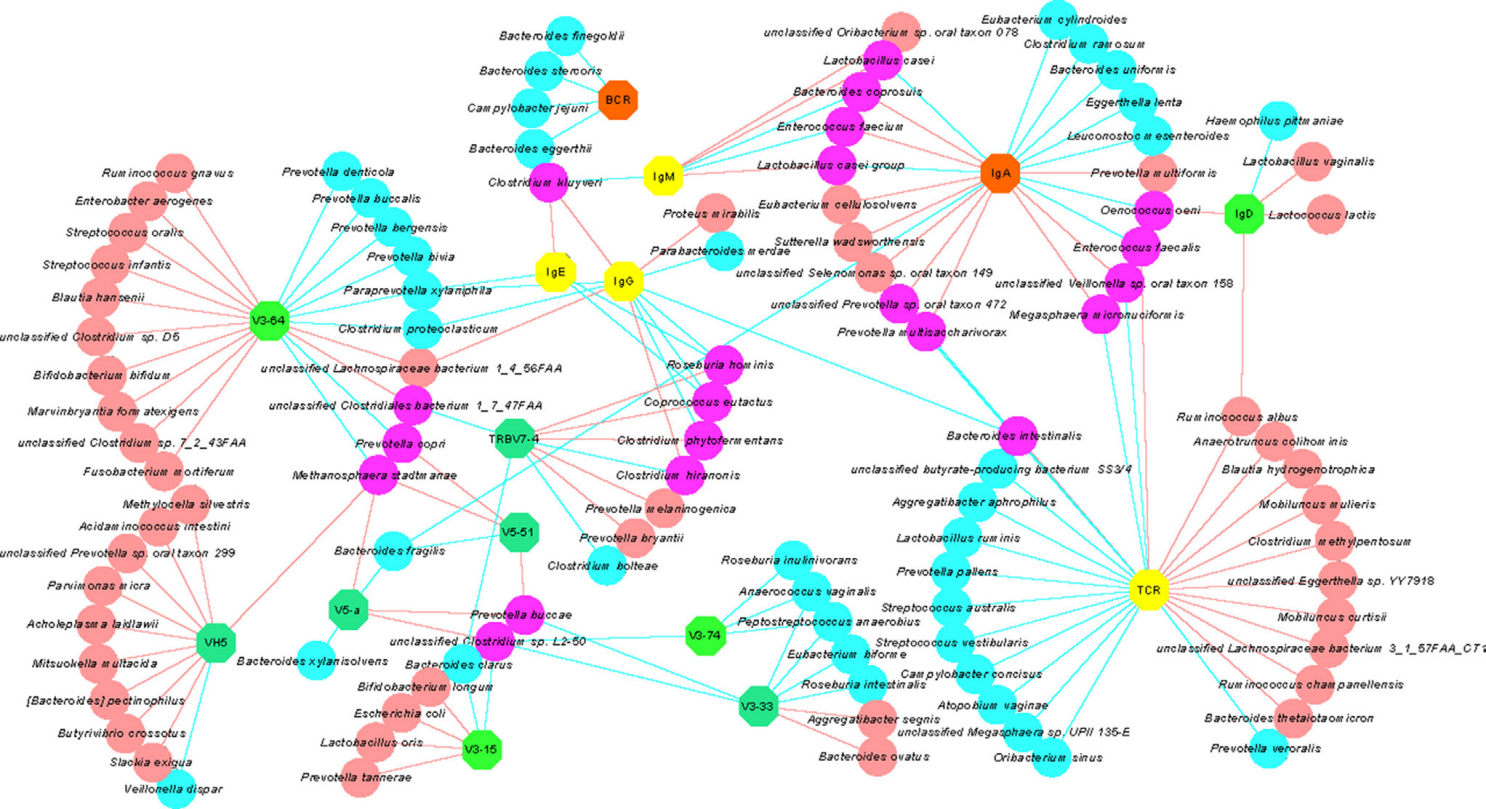
The relationship between immune indexes and bacterial species. Circular, bacterial species significantly associated with a given immune indexes. Abbreviations: Red, positive correlation; blue, negative correlation; purple, positive correlation with one immune index, but negative correlation with the other immune index. Octagon, immune indexes: light green, immune index that significantly differed between A and B subgroups (for example, V3-64); teal-green, immune index that differed significantly between A and B subgroups, as well as between control 1 and control 2 groups (for example, V5-51); yellow, immune index that differed significantly between control 1 and control 2 groups (for example, IgE and T-cell receptor); orange, immune index that did not differ significantly in any comparison for diet pattern (for example, IgA).

In order to examine the association between immune indexes and intestinal microorganisms, a Spearman rank correlation test was conducted to explore associations between bacterial species (with relative abundance >1E-07) and selected immune indexes, including those that were significantly altered after changing the diet or that were associated with a long-term vegetarian diet (Table S11 in Supplementary Material). A network diagram was built to show the correlations (red, positive and blue, negative associations; Figure 7). The symbols of immune indexes were furthermore color-coded based on their association with the diet pattern to allow a multi-dimensional view of our immunological and metagenomic data. Several immune indexes that were significantly changed after the short-term vegetarian diet, including *IGHV5* (*IGHV5-a* and *IGHV5-51*), *IGHV3-15, IGHV3-33, IGHV3-64, IGHV3-74*, and *TRBV7-4*, were positively or negatively associated with a number of bacterial species (Figure 7). The diversity of TCRs, which is negatively associated with long-term vegetarian diet, was positively/negatively associated with large number of species. For examples, the diversity of TCR repertoire was positively associated with control group 1 enriched species such as *Mobiluncus curtisii* and negatively associated with control group 2 enriched species such as *Campylobacter concisus* (Figure 7). Some species negatively associated with the diversity of TCRs, such as *Megasphaera micronuciformis* and *Prevotella multisaccharivorax*, were positively associated with the expression of IgA. In contrast to TCRs, the diversity of BCRs was only negatively associated with a totally different small set of species, of which, *Bacteroides finegoldii* and *Bacteroides stercoris* were both enriched in control group 1.

## Discussion

The dietary pattern and nutrition status can influence the immune system directly, but may also modulate it indirectly by regulating the gut microbiota. Here, we studied the effect of vegetarian diets on the immune repertoires and gut metagenome using high-throughput sequencing technologies. A short-term vegetarian diet did not seem to have any major effect on the diversity of the immune system and the overall composition of the metagenome. A long-term vegetarian diet, however, seemed to be associated with a lower diversity of T-cell repertoire and a microbiota that is less “inflammatory.”

Minor but still significant changes in immune parameters were observed after the short-term vegetarian diet. The usage of certain *IGHV* genes, including *IGHV3-33, IGHV3-64*, and *IGHV3-74*, which have been found in auto-antibodies in systemic lupus erythematosus patients (43), decreased in the study group after changing the diet. The frequency of *IGHV* genes within the *IGHV5* family, which is highly used in the transglutaminase 2-specific auto-antibodies induced by gluten in celiac disease (CD) (45), was, on the other hand, increased at the same time point. However, intriguingly, the expression of these *V* genes showed an opposite trend in the long-term vegetarians, i.e., if a given *V* gene was increased in the short-term vegetarians, for instance *IGHV5-51*, it was reduced in the long-term vegetarians. Thus, short- and long-term vegetarians show opposite effect on the few known autoimmunity associated *IGHV* genes. CD patients have also previously been shown to have an altered gut microbiota with higher levels of *Bacteroides fragilis* (48). However, the expression of *IGHV5-51* was clearly negatively associated with the *B. fragilis* in normal individuals analyzed here. Thus, the short-term vegetarian diet seems to be associated with an increased expression of autoimmune disease (such as CD)-related *V* genes but a reduced abundance of CD related gut bacterial species. This illustrates the complexity of interaction of immunity, microbiota, and diet and their impact on the development of diseases.

The overall pattern of the gut microbiota appears to be quite stable to short-term dietary changes (18). In support of this notion, the 3-month vegetarian diet did not have an effect on gut enterotype or gut microbiota alpha diversity. The beta diversity at the genus or species level, however, decreased in individuals changing to the short-term vegetarian diet. This seems to be contradictory to the recent study that claimed that the structure of microbial community could be altered after a 5-days plant- or animal-based diet (19). It should be noted that the diets in that study was composed of either plant (grains, fruits, and vegetables) or animal products (meats, eggs, and cheese), whereas our short-term vegetarian diet was only devoid of meat and the control group (omnivores) consumed both animal and plant products, which is a more natural setting. Furthermore, the previous study was based on 16S ribosomal RNA gene sequencing, whereas we performed metagenomic shotgun sequencing, which is more comprehensive. Nevertheless, similar to our observation, the alpha diversity actually remained the same after changing the diet in the previous study (19). Moreover, we observed that the abundance of 7 genera and 12 species was significantly changed after the short-term vegetarian diet, which is largely consistent with the previous study (19). For instance, species belonging to *Roseburia* and *Ruminococcus*, which are involved in the digestion of plant polysaccharides, increased after the short-term vegetarian diet. These have previously been shown to decrease after a short-term meat-rich diet (19). Accordingly, several species belonging to the bile-tolerant *Alistipes*, which has previously been associated with a meat-rich diet (19), showed a lower abundance after the short-term vegetarian diet. *Roseburia* and *Ruminococcus* are all producers of the short chain fatty acid butyrate, which has been shown to affect T-cells by inhibiting T-cell proliferation (49) and by stimulating T regulatory cells (Tregs) (50, 51), as well as inhibiting pro-inflammatory cytokines (52). The butyrate-producing bacteria may also have a protective influence on T2D-associated pathogens (25). Moreover, a short-term vegetarian diet could potentially have a beneficial effect on the intestine, since the prevalences of certain probiotic species, including *L. plantarum* and *Streptococcus thermophiles* were increased, whereas the IBD-associated species *Proteus mirabilis* (53) were decreased, in the study group. However, the presence of probiotic species might not necessarily be an indication of a healthy status, since *L. salivarius* was shown to be enriched in patients with rheumatoid arthritis (RA) (54).

There are also a number of bacterial genera/species that showed significant enrichment in long-term vegetarians. *Haemophilus, Neisseria, Aggregatibacter*, and *Veionella* were more prevalent in the long-term vegetarians; these were, according to a recent study, reduced in RA patients as compared to healthy controls (54). The same study found that species within these genera were negatively correlated with the level of C-reactive protein, which is a marker of inflammation. *Haemophilus* was also found to be more prevalent in healthy controls compared to patients with T2D (25). Furthermore, although our cohort is small, it is important to note that long-term vegetarians show a decreased abundance of LPS-related microbial genes (Figure 5), demonstrating a possible anti-inflammatory effect of the long-term vegetarian diet.

The long-term vegetarians also had lower expression levels of IgE, a key immune index for allergy. As both the microbiota (Table S10 in Supplementary Material) and serum IgE level might be associated with BMI (55, 56), we also compared the BMI in different groups and found no difference in our study and control groups. Notably, the TCR diversity was furthermore lower in the long-term vegetarians, which could potentially be due to a dampened overall T-cell response in the gut, resulting in a lower level of both Th1 (proinflammatory) and Th2 (allergic) responses. However, due to the low number of participants in this group the result should be interpreted cautiously. The short-term vegetarians also showed a decreased level of IgE, although not to a significant degree. Thus, the long-term, and to some extent the short-term vegetarian diet, may have a beneficial effect on preventing atopic diseases through regulation of IgE production and modulation of selected gut bacteria. Notably, *Coprococcus eutactus* was negatively associated with IgE level, and increased abundance of *C. eutactus* correlated with alleviated symptoms in 6 months old infants with atopic eczema (57). Further characterization of the bacterial species correlated to IgE level might also be of considerable interest for potential intervention in atopic disease.

Several immune parameters, which did not correlate with a specific diet, nevertheless, showed an association with the abundance of certain gut bacterial species. One of these was IgA, which is the predominant Ig class in the gut and is an important regulator of commensal bacteria (15–17). *Clostridium ramosum* showed a negative correlation with IgA and has previously been reported to correlate with symptoms of metabolic syndrome in women with T2D (28) and found at higher levels in individuals with low bacterial gene richness and obesity (29). *Eggerthella lenta*, also negatively associated with IgA, was reported as a cause of anaerobic spondylodiscitis and was enriched in T2D patients (25). On the other hand, other species with a negative association with IgA, such as the probiotic bacteria *Lactobacillus casei* and *Leuconostoc mesenteroides*, and *B. uniformis* (58) seem to have beneficial effects on the gut microbiota. Furthermore, *B fragilis*, usually is considered as a pathogen and negatively correlated with IgA, produces polysaccharide A, which induces the development of Tregs cells that secrete the anti-inflammatory cytokine interleukin (IL)-10 (59), subsequently inhibiting intestinal inflammation. Expression of IgA was also positively correlated with a number of species. One of these, *Enterococcus faecium*, is an opportunistic pathogen that is resistant to multiple antibiotics (60). Hence, it seems that the presence of IgA in the gut mucosa maintains a homeostasis of a large number of species, including both “good” and “bad” bacteria. Another interesting observation is that the species associated with IgM showed either positive or negative correlation with IgA, while IgG and IgE did not share any species associated with IgA levels, suggesting that the immune response to various gut bacteria may be mediated by different Ig isotypes.

In summary, we have, for the first time, used NGS technology to study the effect of a vegetarian diet on both immunity and gut microbiota simultaneously at a molecular level. We have shown that the interaction of diet, immune system, and the gut microbes is complex. Although vegetarian diets might provide certain benefits to our health through modulation of the immune system and the gut microbiota, there are still many gaps in our understanding of the mechanisms involved and further studies with larger number of individuals will be required.

## Ethics Statement

All study subjects have signed a consent to participate in the study. The study was approved by the institutional review board at the Karolinska Institutet (Reference number: 2013/150-31/2).

## Author Contributions

CZ, AB, KC, GL, CW, YL, HX, JH, and LS performed research and analyzed data. CZ, AB, KC, and QP-H wrote the manuscript. KK and LH reviewed the manuscript. QP-H, JH, LH, and JW designed and supervised research.

## Conflict of Interest Statement

JW is the founder of iCarbonX. All other authors declare no competing interests.
