# Supplementary material for: Impact of a 3-Months Vegetarian Diet on the Gut Microbiota and Immune Repertoire
Source: Front Immunol. 2018 Apr 27;9:908. doi: 10.3389/fimmu.2018.00908 (PMC5934425; doi:10.3389/fimmu.2018.00908)
Supplement: Supplementary file 1 [file image_1.PDF]

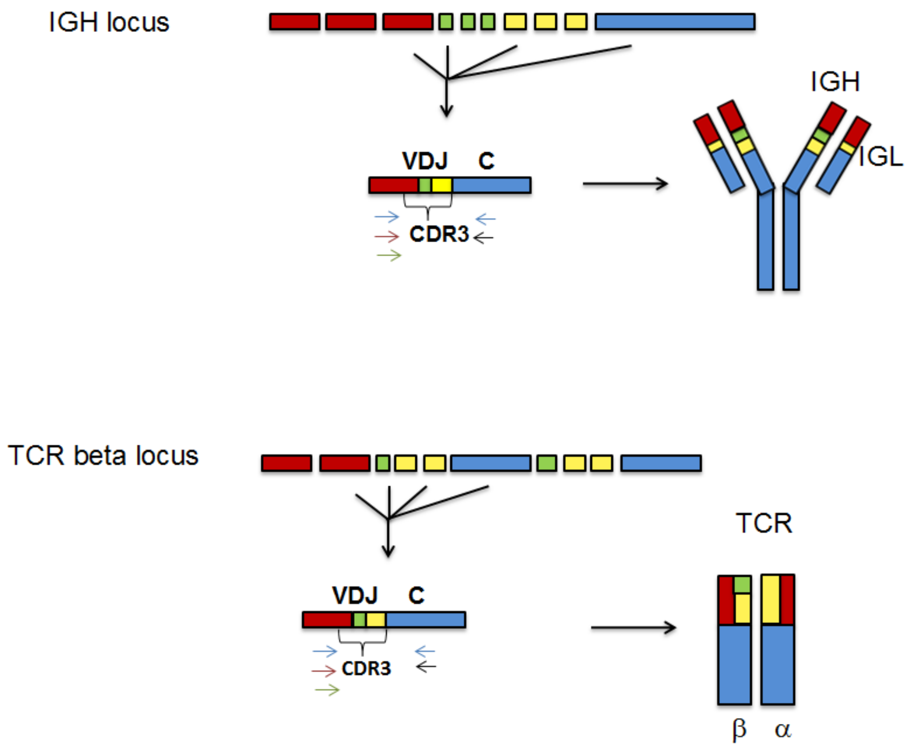

Fig. S1: *Strategy for PCR amplification of the IGH and TCR beta repertoires*. Schematic figures, not to scale, of the *IGH* and *TCRβ* loci before and after V(D)J recombination. Approximate positions of the primers are depicted. A pool of primers specific for the *V* gene families and *C* genes were used. The IG light chain (IGL) at the BCR and TCRα are transcribed from loci not shown in the figure.
