# Supplementary material for: Impact of a 3-Months Vegetarian Diet on the Gut Microbiota and Immune Repertoire
Source: Front Immunol. 2018 Apr 27;9:908. doi: 10.3389/fimmu.2018.00908 (PMC5934425; doi:10.3389/fimmu.2018.00908)
Supplement: Supplementary file 2 [file image_2.PDF]

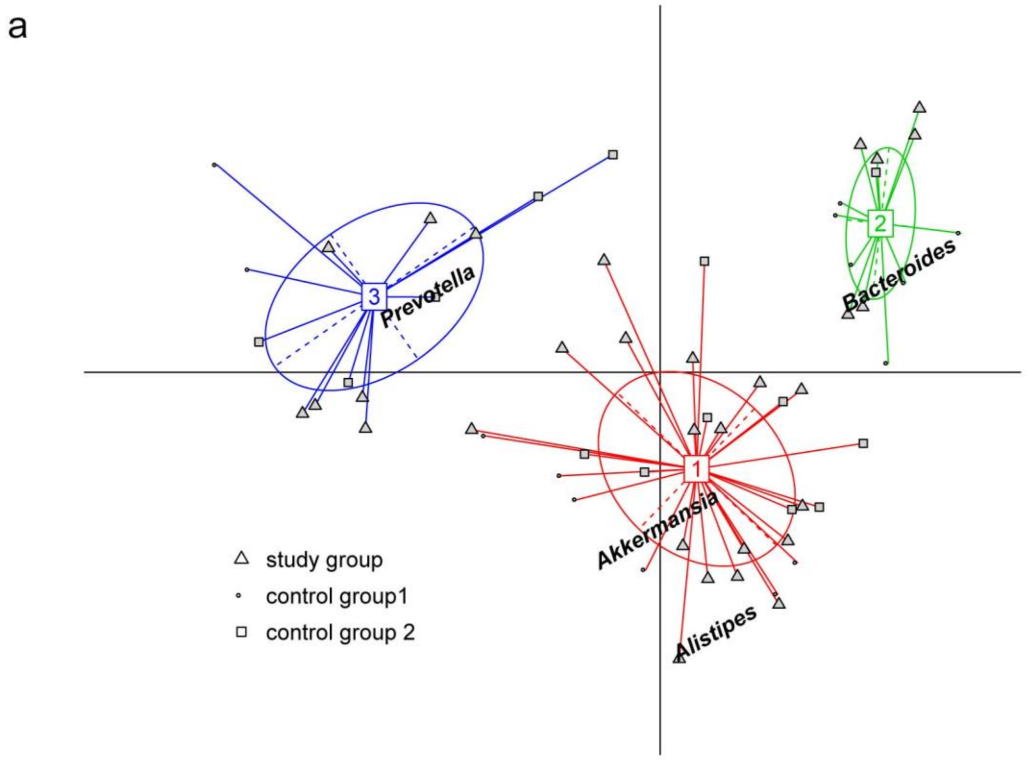

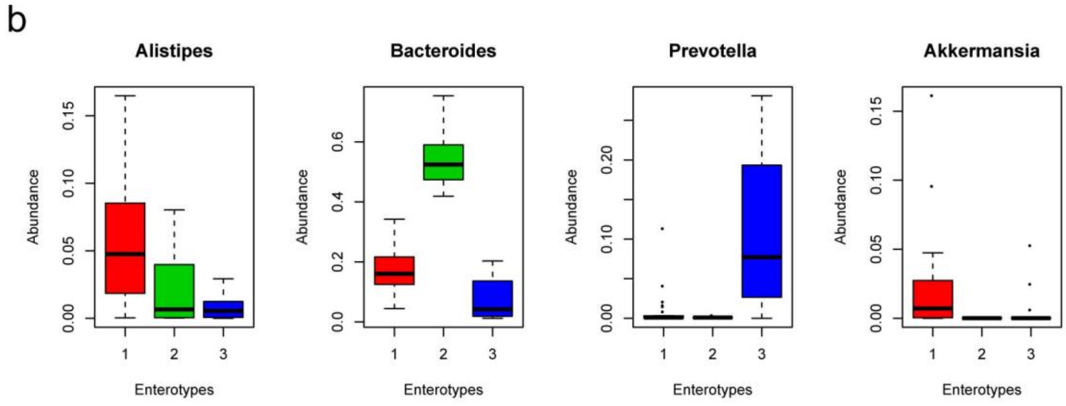

Fig. S2: ***Enterotype*****. (a)** Three enterotypes were identified at the genus level in the samples. **(b)** A principal component analysis (PCA) showed that these three enterotypes were primarily driven by several highly abundant genera, including *Alistipes Bacteroides*, *Prevotella* and *Akkermansia*.
