# Supplementary material for: Impact of a 3-Months Vegetarian Diet on the Gut Microbiota and Immune Repertoire
Source: Front Immunol. 2018 Apr 27;9:908. doi: 10.3389/fimmu.2018.00908 (PMC5934425; doi:10.3389/fimmu.2018.00908)
Supplement: Supplementary file 4 [file image_4.PDF]

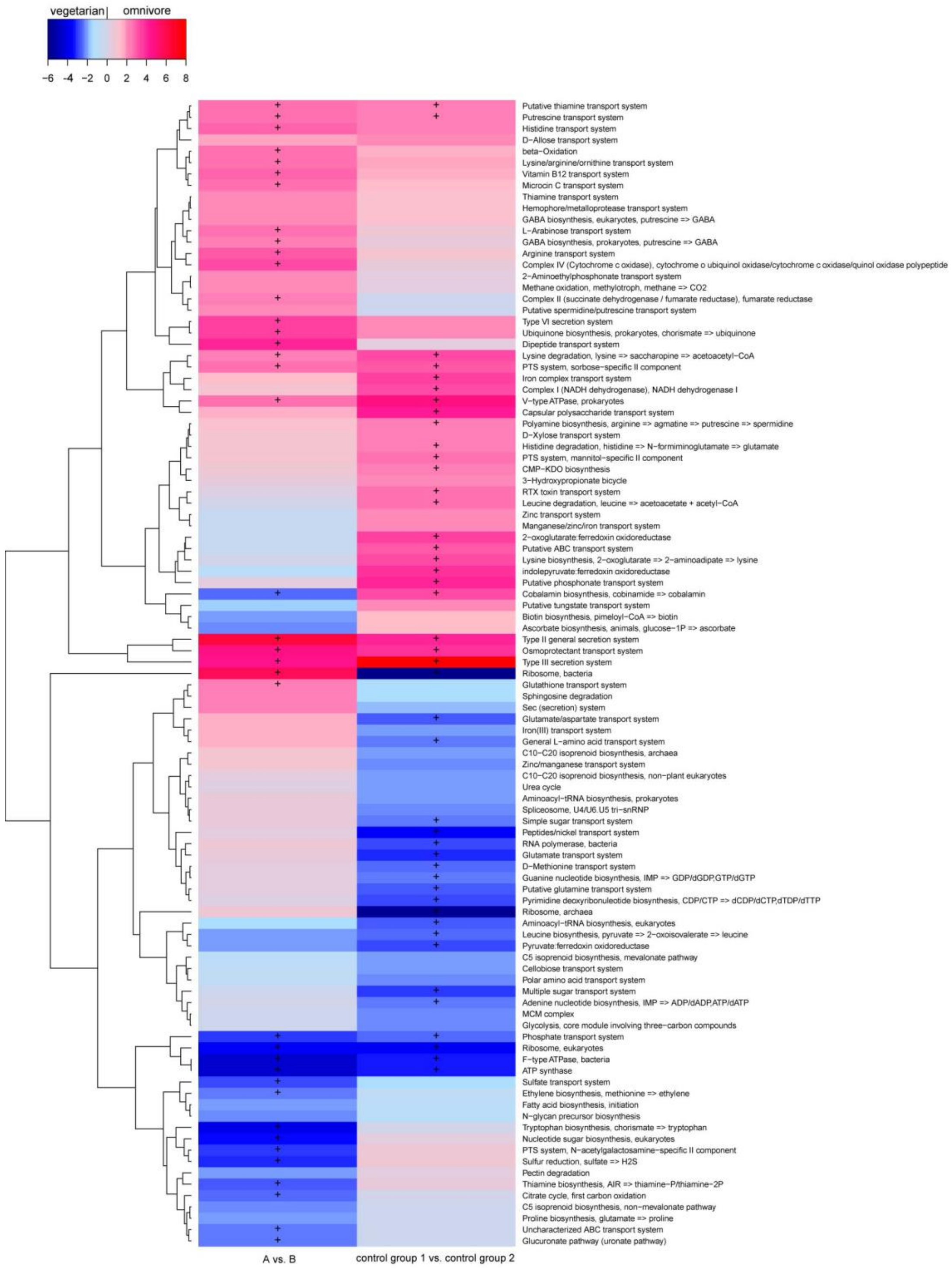

Fig. S4*: KO modules associated with short- and long-term vegetarian diets.* The relative abundances of KO modules were compared between A and B subgroups, between control 1 and control 2 groups and presented as a heatmap. The KO modules with a significant difference in reporter-score are highlighted (+: the reporter score >2.3 or <-2.3; red: enriched in subgroup A or control group 1; blue: enriched in subgroup B or control group 2).
