# Supplementary material for: Impact of a 3-Months Vegetarian Diet on the Gut Microbiota and Immune Repertoire
Source: Front Immunol. 2018 Apr 27;9:908. doi: 10.3389/fimmu.2018.00908 (PMC5934425; doi:10.3389/fimmu.2018.00908)
Supplement: Supplementary file 5 [file image_5.PDF]

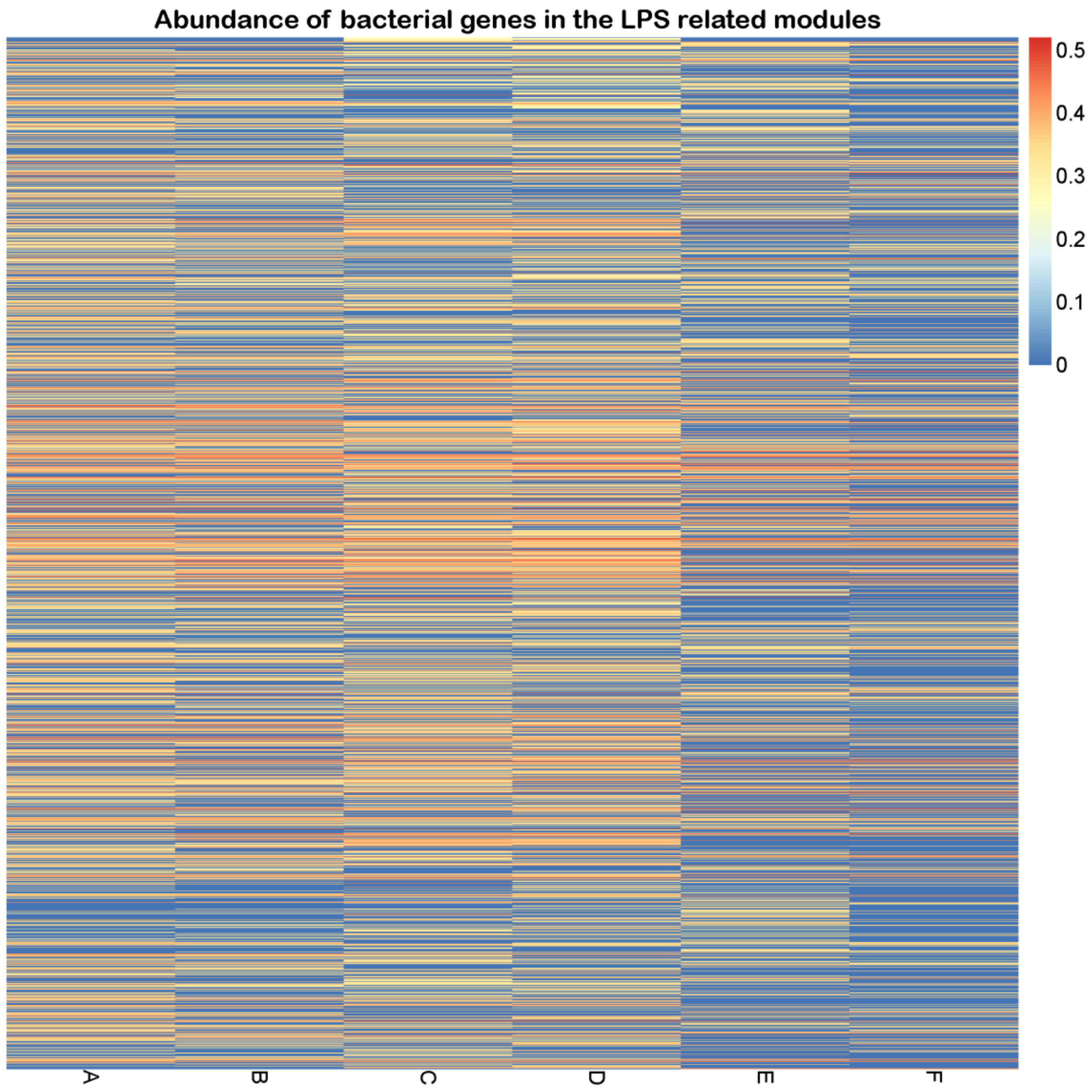

Fig. S5: Abundance of genes in the lipopolysaccharide (LPS) related modules in the different subgroups (heatmap view).
